# Associations between environmental factors and serological Epstein‐Barr virus antibodies in patients with nasopharyngeal carcinoma in South China

**DOI:** 10.1002/cam4.2348

**Published:** 2019-06-26

**Authors:** Ting Zhou, Da‐Wei Yang, Yong‐Qiao He, Wen‐Qiong Xue, Ying Liao, Mei‐Qi Zheng, Yi‐Jing Jia, Lei‐Lei Yuan, Wen‐Li Zhang, Yi‐Xin Zeng, Wei‐Hua Jia

**Affiliations:** ^1^ School of Public Health Sun Yat‐sen University Guangzhou China; ^2^ State Key Laboratory of Oncology in South China, Collaborative Innovation Center for Cancer Medicine, Guangdong Key Laboratory of Nasopharyngeal Carcinoma Diagnosis and Therapy Sun Yat‐sen University Cancer Center Guangzhou China; ^3^ Cancer Center of Guangzhou Medical University Guangzhou China

**Keywords:** cigarette smoking, Epstein‐Barr virus antibodies, herbal tea, nasopharyngeal carcinoma

## Abstract

Epstein‐Barr virus (EBV) reactivation, reflected by aberrantly increased levels of various serological antibodies, has been suggested to be an early indicator of nasopharyngeal carcinoma (NPC) onset and progression. We have previously suggested that certain lifestyle and dietary factors were associated with elevated serological levels of the antibody against various EBV antigens namely VCA, Zta, EBNA1, and oral EBV DNA loads among healthy population. It remains unclear whether these potential environmental factors would also influence EBV serological antibodies in NPC patients. We conducted an epidemiological study to evaluate the associations between such environmental factors and EBV antibody levels among 1701 NPC patients in South China. Pretreatment serums were collected and examined for VCA‐IgA and EA‐IgA by immunoenzymatic assays and antienzyme rate (AER) of EBV DNase‐specific neutralizing antibody. We found that consumption of Canton‐style herbal tea was significantly correlated with increased serological antibody levels of VCA‐IgA and EA‐IgA, with adjusted ORs of 1.35 (95% CI: 1.03‐1.76) and 1.32 (95% CI: 1.01‐1.73), respectively, in the weekly intake frequency stratum, while not related to AER of EBV DNase‐specific neutralizing antibody. Smoking was found to be not only an apparent risk factor for higher antibody levels of AER in stage III‐IV patients (OR = 1.60, 95% CI: 1.11‐2.30), but also associated closely with NPC stage at diagnosis (OR = 2.17, 95% CI: 1.47‐3.22), with dose‐response effects. In conclusion, we found consumption of Canton‐style herbal tea and cigarette smoking were in positive associations with elevated EBV antibodies in NPC patients, which may be of public health significance for the primary prevention of EBV‐associated diseases especially NPC.

## 1 INTRODUCTION

Epstein‐Barr virus (EBV), the first human tumor virus to be discovered^1^ has been estimated to account for more than 120,000 cases of cancer each year.^2^ EBV infection is prevalent in more than 95% of adults worldwide, which occurs typically in childhood and lasts a lifetime. After primary infection, the virus preferentially establishes latent infection in memory B cells and can be periodically reactivated into lytic phase in response to endogenous or environmental stimuli.^3^, ^4^ Such virus reactivation is characterized by active viral replication and release, and aberrantly increased levels of immunoglobulin A (IgA) antibody against various EBV antigens, such as viral capsid antigen (VCA), early antigen (EA), BZLF1 transcription activator protein (Zta) and so on.

Nasopharyngeal carcinoma (NPC) is a rare malignancy worldwide with a strikingly high incidence in Southern China and Southeast Asia^5^ and its etiological association with EBV reactivation has long been established.^6^, ^7^ Evidence from large perspective epidemiological studies has suggested that elevated antibodies could be detected 5‐10 years preceding NPC diagnosis and NPC risk could increase by 20‐30 times in people with elevated levels of EBV antibodies.^8^, ^9^ Several serological EBV antibodies, such as VCA‐IgA, EA‐IgA, and ENBA1‐IgA have served as the effective biomarkers in the screening of NPC in endemic areas for decades.^8^, ^9^, ^10^, ^11^, ^12^, ^13^, ^14^, ^15^, ^16^ Moreover, serological profiles of EBV have been evidenced in clinical practice to be indicative of tumor burden, treatment response and prognosis of NPC patients.^17^, ^18^, ^19^, ^20^ All the above suggested that elevated EBV antibodies may serve as early indicators of NPC onset or progression. Hence, it is of great significance for the primary prevention of NPC to explore the inducing factors that trigger elevated levels of EBV antibodies.

Our previous large‐scale multicenter epidemiological study observed that people in endemic areas of NPC have 2‐5 fold higher risk of EBV reactivation than that in nonendemic areas,^21^, ^22^ which suggested that some genetic or environmental factors in high incidence areas may be implicated in the EBV reactivation. Certain lifestyle and dietary factors, such as cigarette smoking and salted fish consumption, a traditional favorite item in the Cantonese diet, have been well‐established to be risk factors for NPC in endemic areas.^21^, ^23^, ^24^ It is reasonable to assume that such environmental factors may be involved in the pathogenesis of NPC via promoting EBV reactivation. In our previous multicenter epidemiological studies in healthy populations,^21^, ^22^, ^25^ smoking was identified as an apparent risk factor for increased VCA‐IgA, EBNA1‐IgA, Zta‐IgA, and higher oral EBV loads, and consumption of Canon‐style herbal tea was found associated with higher oral EBV loads. Given the different disease status, there may be some differences between patients and healthy people in EBV antibody influencing factors. Since few studies have described in detail the environmental risk factors for EBV antibodies among NPC patients, it is unclear whether some environmental factors would trigger elevated levels IgA antibodies of EBV‐specific antigens.

Previous cohort studies have reported that VCA‐IgA, EA‐IgA, and EBV DNase‐specific was associated with increased NPC risk in NPC endemic areas of South China and Taiwan.^8^, ^9^ VCA‐IgA and EA‐IgA have been widely used as effective biomarkers for screening and auxiliary diagnosis of NPC, due to the high sensitivity of VCA‐IgA and high specificity of EA‐IgA.^14^, ^26^ EBV DNase‐specific neutralizing antibody has been suggested as independent prognostic markers in NPC prognostication.^18^ These three EBV antibodies have been used as the most important clinical indicators for auxiliary diagnosis and monitoring of NPC in Sun Yat‐sun University Cancer Center (SYSUCC) since the 1980s, the largest NPC cancer center with more than 4000 new NPC cases each year. Therefore, we conducted the present epidemiological study in 1701 NPC patients without treatment from Guangdong province, trying to identify potential environmental risk factors related to VCA‐IgA, EA‐IgA and EBV DNase‐specific neutralizing antibody.

## 2 MATERIALS AND METHODS

### 2.1 Subjects

**Figure 1:**
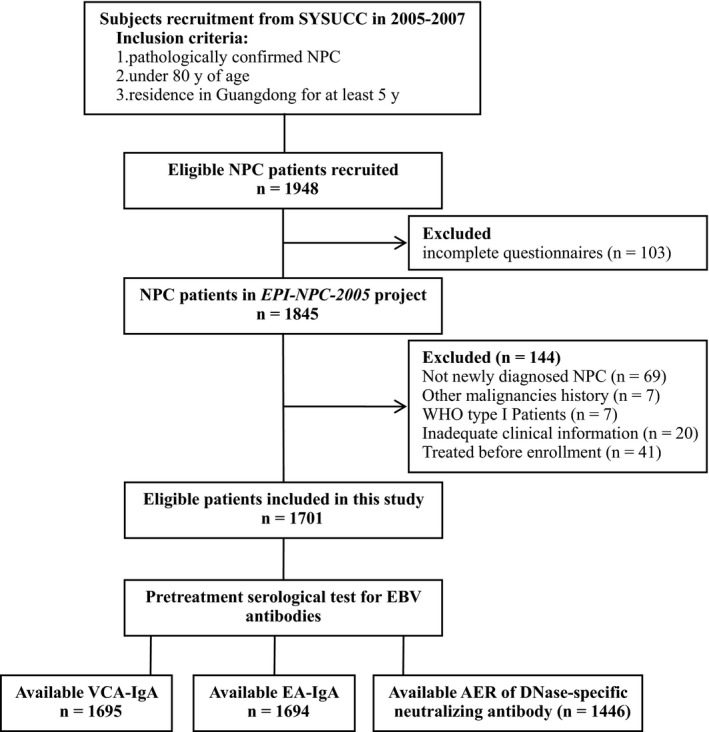
Flow chart of subject inclusion. NPC, nasopharyngeal carcinoma; SYSUCC, Sun Yat‐sun University Cancer Center; VCA‐IgA, immunoglobulin A antibodies against viral capsid antigen; EA‐IgA, immunoglobulin A antibodies against early antigen; AER, anti‐enzyme rate.

The present hospital‐based case‐only study was conducted on the basis of a case‐control study named as *EPI‐NPC‐2005* project conducted during October 2005 and October 2007, which was designed to assess the interaction between EBV, environmental factors and genetic determinants in the pathogenesis and progression of NPC. Briefly, NPC patients were recruited from Sun Yat‐sun University Cancer Center (SYSUCC), Guangzhou, China, according to the following criteria: (a) pathologically confirmed NPC; (b) under 80 years of age; (c) residence in Guangdong Province for at least 5 years. Among 1948 eligible patients enrolled, 1845 patients (94.7%) completed the interviews. In this study, we further excluded those with previous diagnoses of NPC or other malignancies, those having received radiotherapy or chemotherapy before blood sampling, those with WHO type I NPC, and those without sufficient medical record information available. Finally, a total of 1701 eligible patients were included in this study. Flow chart of patient inclusion is shown in Figure 1. Written informed consent was obtained from each participant at enrollment. The study was approved by the Ethics Committee of the Sun Yat‐Sen University Cancer Center.

### 2.2 Data collection

Personal information was collected using a structured questionnaire though face‐to‐face interviews by trained staffs. The collected information mainly included demographics (age, gender, education level), lifestyle habits (smoking behavior, alcohol drinking, tea drinking, consumption of preserved vegetables, salted fish, Canton‐style herbal tea, and slow‐cooked soup, etc), and family history of malignancy and NPC among first‐degree relatives. Medical information including TNM stage and WHO pathology type and other relevant information was retrieved from the hospital information system (HIS) of SYSUCC.

### 2.3 Serologic assay for VCA‐IgA and EA‐IgA antibodies

A quantity of 5‐10 mL pretreatment blood sample was collected from each eligible patient. Serum VCA‐IgA and EA‐IgA antibody levels were measured using commercial kit (Zhongshan Bio‐tech Co Ltd, Zhongshan City, China) following an immunoenzymatic procedure as described in our previous study^21^, ^27^: (a) Cell smears were prepared from the B95‐8 cell line for VCA‐IgA and the Raji cell line for EA‐IgA, and fixed with acetone in the wells of slides; (b) a series of gradient‐diluted serum was added to the wells; (c) the slides were incubated for 30 minutes at 37°C and washed with phosphate buffered saline (PBS) three times; (d) peroxidase‐conjugated antihuman IgA antibody was added and incubated (1 hour at 37°C) and then washed with PBS thrice; (e) aminoethylcarbazole solution and hydrogen peroxide (H_2_O_2_) were added for 15 minutes. The slides were then examined under the optical microscope and brown staining in more than 15% of the cells was considered as positive. The antibody titrations of VCA‐IgA or EA‐IgA were expressed as the reciprocal of the highest dilution with positive brown staining. All blood tests were conducted by the same technician in the same laboratory. The cutoff adopted in this study was based on the median titer among all patients, 1:320 for VCA and 1:40 for VCA‐IgA, respectively.

### 2.4 Serologic assay for AER of EBV DNase‐specific neutralizing antibody

The assay for serologic antienzyme rate (AER) of EBV DNase‐specific neutralizing antibody was carried out as previously described by Xu et al.^17^ Briefly, Raji cells were cultured with croton oil and n‐butanoic acid to induce EBV‐specific DNase production. After 48‐hours incubation, the Raji cells were harvested and washed with PBS. Extraction buffer containing Mg^2+^‐adenosine triphosphate, KCl, dithiothreitol, and Tris‐HCl was added and the mixture was frozen and thawed four times. After centrifugation, the supernatant was mixed with the sample serum and stayed at room temperature for 10 minutes. After addition of the reaction buffer containing Tris‐HCl, ^3^H‐DNA, MgCl_2_, and β‐mercaptoethanol, the mixture was incubated at 37°C for 20 minutes and the reaction was blocked with trichloroacetic acid. After centrifugation, the supernatant was transferred to a filter paper. The dried filter paper was put into a scintillation bottle with scintillation liquid containing paraphenylene phenyloxasole and 2,5‐diphenyloxasole, and was analysed by an automatic liquid scintillation counter (LS6500; Beckman Coulter, Brea, CA). Double distilled water was used as negative control. The result obtained from the liquid scintillation counter was expressed as the counts per minute (CPM). AER was calculated by the following formula: AER = [1 – (sample serum CPM/ddH_2_O CPM)] × 100%. The cutoff value to separate low or high level of AER was 50% in this study.

### 2.5 Statistical analyses

All serological EBV antibody markers were dichotomized into binary variables according to their corresponding median values in the whole population. Univariate and multivariate unconditional logistic regressions were performed to assess the associations of EBV serum antibody levels with potential lifestyle factors. Linear trend tests were carried out by treating ordered categorical variables as continuous variables. The adjustments in multivariate analysis included age, sex, education, clinical stage, and all other environmental factors. Subgroup analyses were performed to evaluate the relationship between smoking and EBV antibodies. All statistical tests were two‐sided and *P* < 0.05 was considered statistically significant. Analyses were performed using Stata version10.0 (Stata Corp, College Station, TX).

## 3 RESULTS

### 3.1 Patients characteristics

The clinicopathologic characteristics of the study subjects are summarized in Table 1. In total, 1701 NPC patients were included in this study, 1246 males and 455 females. The subjects ranged in age from 13 to 80, with a median age of 45. Patients with WHO type III NPC accounted for 95% of the population. About 80% of the subjects were diagnosed with advanced NPC (stage III‐IV). Serological antibody titers against VCA‐IgA were available in 1695 (99.65%) patients and EA‐IgA in 1694 (99.59%) patients. The AER of DNase‐specific neutralizing antibody was available in 1446 (85.01%) patients.

**Table 1:** Baseline characteristics of 1701 NPC patients

Characteristics	No. of patients	%
Age at diagnosis (years)		
Median (range)	45 (13‐80)	
≤30	118	6.94
31‐40	432	25.40
41‐50	571	33.57
51‐60	405	23.81
≥61	175	10.29
Sex		
Female	455	26.75
Male	1246	73.25
Education		
Primary school or less	397	23.44
High school	1039	61.33
University or more	258	15.23
WHO Histology type		
II	87	5.11
III	1614	94.89
Overall stage		
I	51	3.00
II	277	16.28
III	884	51.97
IV	489	28.75
T stage		
T0‐T1	106	6.23
T2	407	23.93
T3	813	47.80
T4	375	22.05
N stage		
N0	401	23.57
N1	685	40.27
N2	494	29.04
N3	121	7.11
M stage		
M0	1657	97.41
M1	44	2.59
Family history of NPC		
No	1415	83.38
Yes	282	16.62
Family history of tumor		
No	995	58.63
Yes	702	41.37
Cigarette smoking		
Never smoker	767	45.38
Ex‐smoker	443	26.21
Current smoker	480	28.40
Alcohol drinking		
Nondrinker	969	57.51
≤1 drink per day	406	24.09
>1 drink per day	310	18.40
Tea consumption		
Less than monthly	595	35.59
Monthly	284	16.99
Weekly or more	793	47.43
Salted fish intake		
Less than monthly	1359	80.37
Monthly	183	10.82
Weekly or more	149	8.81
Preserved vegetable intake		
Less than monthly	1219	71.92
Monthly	253	14.93
Weekly or more	223	13.16
Herbal tea intake		
Less than monthly	434	25.68
Monthly	669	39.59
Weekly or more	587	34.73
Canton soup intake		
Less than monthly	239	14.18
Monthly	192	11.39
Weekly or more	1254	74.42

### 3.2 EBV antibodies and demographic and clinicopathologic characteristics

Among demographic and clinicopathologic factors, age was found to be linearly correlated with antibody levels of VCA‐IgA and EA‐IgA, but not AER of DNase‐specific neutralizing antibody. Higher clinical stage, higher T stage or higher N stage suggested higher levels of VCA‐IgA, EA‐IgA and AER. In addition, WHO type‐III NPCs were associated with high level of AER compared with WHO type II NPCs. No significant associations were observed between sex, education, BMI, and family history of tumor or NPC and any of the three EBV antibodies (Table 2).

**Table 2:** Associations of demographic and clinicopathologic characteristics with EBV antibodies in 1701 NPC patients

Variables	VCA‐IgA		EA‐IgA		AER	
	Low/High^a^	OR (95% CI) b	Low/High^a^	OR (95% CI) b	Low/High^a^	OR (95% CI)^b^
Age (years)						
≤30	67/51	1.00 (reference)	76/42	1.00 (reference)	56/40	1.00 (reference)
31‐40	206/224	1.45 (0.96‐2.21)	242/188	1.46 (0.95‐2.24)	182/192	1.52 (0.96‐2.42)
41‐50	257/311	1.62 (1.07‐2.44)*	315/251	1.53 (1.01‐2.34)*	246/230	1.33 (0.84‐2.11)
51‐60	179/226	1.66 (1.08‐2.55)*	204/201	1.88 (1.21‐2.93)**	179/169	1.25 (0.78‐2.01)
≥61	71/103	1.83 (1.11‐3.01)*	88/87	1.82 (1.10‐3.02)*	67/85	1.61 (0.93‐2.77)
*P*_‐trend_ c		0.027		0.007		0.687
Sex						
Female	219/234	1.00 (reference)	253/199	1.00 (reference)	181/201	1.00 (reference)
Male	561/681	1.08 (0.86‐1.35)	672/570	0.99 (0.79‐1.24)	549/515	0.81 (0.64‐1.04)
BMI						
<18.5	69/73	1.00 (reference)	80/62	1.00 (reference)	58/70	1.00 (reference)
18.5‐23.9	405/458	1.09 (0.76‐1.57)	466/395	1.11 (0.77‐1.60)	398/374	0.86 (0.59‐1.27)
≥24.0	207/275	1.31 (0.89‐1.94)	255/228	1.18 (0.80‐1.75)	216/210	0.92 (0.61‐1.39)
*P*_‐trend_ c		0.085		0.379		0.972
Education						
Primary school or less	171/223	1.00 (reference)	204/190	1.00 (reference)	145/181	1.00 (reference)
High school	468/568	1.04 (0.81‐1.35)	566/469	1.03 (0.80‐1.33)	452/439	0.83 (0.63‐1.10)
University or more	138/120	0.86 (0.61‐1.21)	150/108	1.04 (0.73‐1.46)	129/94	0.69 (0.48‐1.01)
*P*_‐trend_ c		0.436		0.834		0.055
WHO histology						
II	42/44	1.00 (reference)	52/34	1.00 (reference)	41/24	1.00 (reference)
III	738/871	1.07 (0.69‐1.66)	873/735	1.25 (0.79‐1.96)	689/692	1.71 (1.01‐2.89)*
Clinical stage						
I‐II	206/121	1.00 (reference)	230/97	1.00 (reference)	186/102	1.00 (reference)
III‐IV	574/794	2.27 (1.76‐2.92)***	695/672	2.25 (1.73‐2.93)***	544/614	2.01 (1.53‐2.63)***
T stage						
T0‐T2	277/234	1.00 (reference)	323/187	1.00 (reference)	262/178	1.00 (reference)
T3‐T4	503/681	1.56 (1.26‐1.92)***	602/582	1.65 (1.33‐2.05)***	468/538	1.65 (1.31‐2.08)***
N stage						
N0‐N1	565/518	1.00 (reference)	642/440	1.00 (reference)	490/437	1.00 (reference)
N2‐N3	215/397	2.03 (1.65‐2.49)***	283/329	1.72 (1.40‐2.10)***	240/279	1.31 (1.05‐1.63)*
Family history of NPC						
No	656/754	1.00 (reference)	780/628	1.00 (reference)	609/592	1.00 (reference)
Yes	123/158	1.13 (0.87‐1.46)	145/137	1.19 (0.91‐1.54)	120/122	1.07 (0.81‐1.42)
Family history of tumor						
No	448/545	1.00 (reference)	534/458	1.00 (reference)	412/420	1.00 (reference)
Yes	331/367	0.94 (0.77‐1.15)	391/307	0.93 (0.77‐1.14)	317/294	0.94 (0.76‐1.17)

Abbreviations: VCA‐IgA, immunoglobulin A antibodies against viral capsid antigen; EA‐IgA, immunoglobulin A antibodies against early antigen; AER, antienzyme rate.

a For VCA‐IgA titers, a low level refers to titers < 1:320 and a high EBV level refers to ≥ 1:320; for EA‐IgA titers, a low level refers to titers < 1:40 and a high EBV level refers to ≥ 1:40; for AER, a low level refers to < 50% and a high EBV level refers to ≥ 50%.

b Adjusted for age (continuous variable), sex, education, clinical stage.

c Linear trends tests were performed by treating ordered categorical variables as continuous variables.

* *P* < 0.05,

** *P *< 0.01,

*** *P* < 0.001.

### 3.3 EBV antibodies and Canton‐style herbal tea consumption

Among all evaluated lifestyle factors, consumption of Canton‐style herbal tea was found the only risk factor for elevated serological antibody levels of VCA‐IgA and EA‐IgA. In univariate analysis, patients who consumed herbal tea weekly were at increased risk for higher VCA‐IgA compared with those consuming less than monthly, with ORs of 1.38 (95% CI: 1.07‐1.77) and 1.31 (95% CI: 1.02‐1.69) for VCA‐IgA and EA‐IgA respectively. When adjusted for age, sex, education level, clinical stage, and other lifestyle factors, the associations remained robust, with adjusted ORs of 1.35 (95% CI: 1.03‐1.76) and 1.32 (95% CI: 1.01‐1.73) for VCA‐IgA and EA‐IgA respectively. However, no association was observed between herbal tea intake and AER of DNase‐specific neutralizing antibody (Table 3).

**Table 3:** Associations of lifestyle risk factors with EBV antibodies in 1701 NPC patients

Variables	VCA‐IgA			EA‐IgA			AER		
	Low/High^a^	OR (95% CI)^b^	OR (95% CI) c	Low/High^a^	OR (95% CI)^b^	OR (95% CI)^c^	Low/High^a^	OR (95% CI)^b^	OR (95% CI)^c^
Cigarette smoking									
Never smoker	375/388	1.00 (reference)	1.00 (reference)	435/327	1.00 (reference)	1.00 (reference)	336/314	1.00 (reference)	1.00 (reference)
Ex‐smoker	201/240	1.15 (0.91‐1.46)	1.06 (0.78‐1.44)	242/199	1.09 (0.86‐1.39)	1.02 (0.75‐1.38)	192/173	0.96 (0.75‐1.25)	1.16 (0.83‐1.61)
Current smoker	199/281	1.36 (1.08‐1.72)**	1.12 (0.82‐1.52)	243/237	1.30 (1.03‐1.63)*	1.02 (0.75‐1.39)	198/223	1.21 (0.94‐1.54)	1.36 (0.97‐1.89)
*P*_‐trend_ d		0.008	0.421		0.028	0.817		0.167	0.064
Alcohol drinking									
Nondrinker	450/518	1.00 (reference)	1.00 (reference)	528/438	1.00 (reference)	1.00 (reference)	407/418	1.00 (reference)	1.00 (reference)
≤1 drink per day	189/214	0.98 (0.78‐1.24)	0.89 (0.68‐1.15)	231/173	0.90 (0.71‐1.14)	0.86 (0.66‐1.11)	189/163	0.84 (0.65‐1.08)	0.78 (0.59‐1.03)
>1 drink per day	135/173	1.11 (0.86‐1.44)	0.89 (0.67‐1.20)	159/149	1.13 (0.87‐1.46)	0.99 (0.74‐1.32)	128/126	0.96 (0.72‐1.27)	0.86 (0.63‐1.19)
*P*_‐trend_ d		0.499	0.377		0.570	0.764		0.498	0.202
Tea consumption									
Less than monthly	284/311	1.00 (reference)	1.00 (reference)	323/272	1.00 (reference)	1.00 (reference)	252/256	1.00 (reference)	1.00 (reference)
Monthly	142/140	0.90 (0.68‐1.20)	0.87 (0.65‐1.17)	166/116	0.83 (0.62‐1.11)	0.82 (0.60‐1.11)	127/125	0.97 (0.72‐1.31)	1.04 (0.76‐1.43)
Weekly or more	339/450	1.21 (0.98‐1.50)	0.89 (0.67‐1.20)	419/370	1.05 (0.85‐1.30)	0.98 (0.77‐1.25)	336/324	0.95 (0.75‐1.20)	1.02 (0.78‐1.32)
*P*_‐trend_ d		0.064	0.199		0.596	0.872		0.660	0.910
Salted fish intake									
Less than monthly	635/720	1.00 (reference)	1.00 (reference)	762/592	1.00 (reference)	1.00 (reference)	596/561	1.00 (reference)	1.00 (reference)
Monthly or more	140/190	1.20 (0.94‐1.53)	1.14 (0.87‐1.50)	159/171	1.38 (1.09‐1.76)**	1.26 (0.96‐1.65)	130/151	1.23 (0.95‐1.60)	1.15 (0.86‐1.54)
Preserved vegetable intake									
Less than monthly	565/652	1.00 (reference)	1.00 (reference)	684/532	1.00 (reference)	1.00 (reference)	532/502	1.00 (reference)	1.00 (reference)
Monthly or more	212/260	1.06 (0.85‐1.32)	0.92 (0.72‐1.17)	239/233	1.25 (1.01‐1.55)*	1.06 (0.84‐1.35)	196/211	1.14 (0.91‐1.44)	0.99 (0.76‐1.28)
Herbal tea intake									
Less than monthly	218/215	1.00 (reference)	1.00 (reference)	247/186	1.00 (reference)	1.00 (reference)	191/174	1.00 (reference)	1.00 (reference)
Monthly	310/358	1.17 (0.92‐1.49)	1.17 (0.91‐1.52)	377/290	1.02 (0.80‐1.30)	1.02 (0.79‐1.32)	285/282	1.09 (0.83‐1.41)	1.09 (0.83‐1.45)
Weekly or more	247/336	1.38 (1.07‐1.77)*	1.35 (1.03‐1.76)*	293/290	1.31 (1.02‐1.69)*	1.32 (1.01‐1.73)*	249/255	1.12 (0.86‐1.47)	1.11 (0.83‐1.48)
*P*_‐trend_ d		0.011	0.034		0.024	0.035		0.405	0.478
Canton soup intake									
Less than monthly	120/118	1.00 (reference)	1.00 (reference)	136/102	1.00 (reference)	1.00 (reference)	106/88	1.00 (reference)	1.00 (reference)
Monthly	86/106	1.25 (0.86‐1.84)	1.09 (0.73‐1.63)	98/94	1.28 (0.87‐1.87)	1.14 (0.76‐1.70)	76/84	1.33 (0.87‐2.03)	1.30 (0.83‐2.01)
Weekly or more	568/681	1.22 (0.92‐1.61)	1.14 (0.85‐1.54)	680/568	1.11 (0.84‐1.47)	1.06 (0.78‐1.42)	542/537	1.19 (0.88‐1.62)	1.22 (0.88‐1.69)
*P*_‐trend_ d		0.222	0.396		0.677	0.954		0.409	0.344

Abbreviations: VCA‐IgA, immunoglobulin A antibodies against viral capsid antigen; EA‐IgA, immunoglobulin A antibodies against early antigen; AER, antienzyme rate.

a For VCA‐IgA titers, a low level refers to titers < 1:320 and a high EBV level refers to ≥ 1:320; for EA‐IgA titers, a low level refers to titers < 1:40 and a high EBV level refers to ≥ 1:40; for AER, a low level refers to < 50% and a high EBV level refers to ≥ 50%.

b Univariate analysis.

c Adjusted for age (continuous variable), sex, education level, clinical stage, smoking status, drinking, tea consumption, salted fish intake, preserved vegetable intake, herbal tea intake, Canton soup intake.

d Linear trends tests were performed by treating ordered categorical variables as continuous variables.

* *P* < 0.05.

** *P* < 0.01.

We further performed subgroup analyses to assess whether the associations of herbal tea consumption with VCA‐IgA and EA‐IgA antibodies differed between patients with different clinical characteristics. Positive associations were observed between herbal tea consumption and VCA‐IgA in those aged 40 or above, women, those with high school education or less, and those with family history of NPC or other tumors rather than in their counterparts. And herbal tea consumption was also found associated with elevated EA‐IgA in those aged 40 or above, those with high school education or less, and those with a family history of tumors instead of their counterparts (Table S1).

### 3.4 EBV antibodies and cigarette smoking

Smoking status was found correlated with higher antibody levels of VCA‐IgA and EA‐IgA in univariate analysis, with ORs of 1.36 (95% CI: 1.08‐1.72) and 1.30 (95% CI: 1.03‐1.63) respectively, but the associations were eliminated with adjustment for age, sex, education, clinical stage and other lifestyle factors in multivariate analysis (Table 3). To further explore association of cigarette smoking with EBV antibodies, we performed further subgroup analyses according to age at smoking initiation, smoking duration, pack‐years of smoking, and type of smoking (inhale or not). However, no significant associations were obtained between all smoking subgroups and any of the three EBV antibodies in multivariate analysis, except for the type of smoking with AER. Interestingly, noninhaled smoking was associated with higher level of AER (OR = 1.65, 95% CI: 1.12‐2.43), whereas inhaled‐smoking was not (Table 4). Stratified analysis was further performed according to the clinical staging, early NPC (stage I‐II) and advanced NPC (stage III‐IV). Positive associations between smoking and AER were observed among advanced NPC patients but not early patients. Current smokers and patients with advanced NPC were at increased risk of higher AER, with OR of 1.60 (95% CI: 1.11‐2.30) compared with never smokers when adjusted for potential confounders. Smokers with longer smoking history, greater cumulative amount of smoking (pack‐years), and noninhaled type of smoking tended to exhibit higher AER among patients with advanced NPC (Table 5). No association was found between smoking and either VCA‐IgA or EA‐IgA in either patients with early NPC or advanced NPC (data not shown).

**Table 4:** Subgroup analysis of the associations between smoking and EBV antibodies in 1701 NPC patients

Smoking status	VCA‐IgA			EA‐IgA			AER		
	Low/High^a^	OR (95% CI) b	OR (95% CI) c	Low/High^a^	OR (95% CI)^b^	OR (95% CI) c	Low/High^a^	OR (95% CI)^b^	OR (95% CI) c
Initial age of smoking (years)									
Never	375/388	1.00 (reference)	1.00 (reference)	435/327	1.00 (reference)	1.00 (reference)	336/314	1.00 (reference)	1.00 (reference)
≥20	199/253	1.23 (0.97‐1.55)	1.08 (0.80‐1.47)	242/210	1.15 (0.91‐1.46)	1.01 (0.74‐1.37)	193/191	1.06 (0.82‐1.36)	1.27 (0.92‐1.76)
<20	201/269	1.29 (1.03‐1.63)*	1.10 (0.81‐1.50)	243/227	1.24 (0.99‐1.57)	1.04 (0.76‐1.42)	198/205	1.11 (0.86‐1.42)	1.23 (0.88‐1.71)
*P*_‐trend_ d		0.022	0.560		0.058	0.798		0.412	0.270
Smoking duration (years)									
Never	375/388	1.00 (reference)	1.00 (reference)	435/327	1.00 (reference)	1.00 (reference)	336/314	1.00 (reference)	1.00 (reference)
<25	204/241	1.06 (0.83‐1.34)	1.00 (0.74‐1.37)	236/175	0.99 (0.77‐1.26)	0.93 (0.68‐1.27)	184/161	0.94 (0.72‐1.22)	1.16 (0.83‐1.62)
≥25	190/267	1.43 (1.14‐1.80)**	1.14 (0.83‐1.58)	242/249	1.37 (1.09‐1.72)**	1.07 (0.77‐1.47)	200/223	1.19 (0.93‐1.52)*	1.39 (0.99‐1.97)
*P*_‐trend_ d		0.003	0.410		0.011	0.665		0.198	0.057
Cumulative amount (pack‐years)									
Never	375/388	1.00 (reference)	1.00 (reference)	435/327	1.00 (reference)	1.00 (reference)	336/314	1.00 (reference)	1.00 (reference)
<30	168/210	1.12 (0.90‐1.39)	1.02 (0.76‐1.36)	319/259	1.08 (0.87‐1.34)	0.98 (0.73‐1.31)	255/239	1.00 (0.79‐1.27)	1.22 (0.89‐1.67)
≥30	222/303	1.60 (1.23‐2.09)**	1.30 (0.91‐1.85)	157/168	1.42 (1.10‐1.85)**	1.09 (0.77‐1.56)	128/147	1.28 (0.93‐1.63)	1.38 (0.94‐2.01)
*P*_‐trend_ d		0.001	0.163		0.012	0.624		0.211	0.094
Inhaled smoking or not									
Never	375/388	1.00 (reference)	1.00 (reference)	435/327	1.00 (reference)	1.00 (reference)	336/314	1.00 (reference)	1.00 (reference)
Noninhaled	105/124	1.14 (0.85‐1.53)	1.01 (0.70‐1.44)	125/104	1.11 (0.82‐1.491)	1.00 (0.70‐1.44)	83/107	1.38 (1.00‐1.91)**	1.65 (1.12‐2.43)*
Inhaled	274/353	1.25 (1.01‐1.54)*	1.02 (0.76‐1.37)	331/296	1.19 (0.96‐1.47)	0.98 (0.72‐1.32)	275/264	1.03 (0.82‐1.29)	1.20 (0.87‐1.65)
*P*_‐trend_ d		0.042	0.902		0.108	0.864		0.765	0.451

Abbreviations: VCA‐IgA, immunoglobulin A antibodies against viral capsid antigen; EA‐IgA, immunoglobulin A antibodies against early antigen; AER, antienzyme rate.

a For VCA‐IgA titers, a low level refers to titers < 1:320 and a high EBV level refers to ≥ 1:320; for EA‐IgA titers, a low level refers to titers < 1:40 and a high EBV level refers to ≥ 1:40; for AER, a low level refers to < 50% and a high EBV level refers to ≥ 50%.

b Univariate analysis.

c Adjusted for age (continuous variable), sex, education level, clinical stage, drinking, tea consumption, salted fish intake, preserved vegetable intake, herbal tea intake, Canton soup intake.

d Linear trends tests were performed by treating ordered categorical variables as continuous variables.

* *P* < 0.05.

** *P* < 0.01.

**Table 5:** Associations between smoking and EBV DNase‐specific neutralizing antibody by clinical stage

Smoking status	I‐II stage		III‐IV stage		*P*‐interaction^d^
	Low/High^a^	OR (95% CI)^b^	Low/High^a^	OR (95% CI)^b^	
Cigarette smoking					
Never smoker	92/58	1.00 (reference)	244/256	1.00 (reference)	
Ex‐smoker	53/26	0.91 (0.45‐1.88)	139/147	1.26 (0.87‐1.83)	
Current smoker	38/18	0.69 (0.30‐1.57)	160/205	1.60 (1.11‐2.30)*	0.094
*P*_‐trend_ c		0.388		0.012	
Initial age of smoking (years)					
Never	92/58	1.00 (reference)	244/256	1.00 (reference)	
≥20	52/24	0.77 (0.37‐1.59)	141/167	1.49 (1.03‐2.15)*	
<20	40/20	0.88 (0.39‐1.96)	158/185	1.37 (0.95‐1.98)	0.236
*P*_‐trend_ c		0.716		0.149	
Smoking duration (years)					
Never	92/58	1.00 (reference)	244/256	1.00 (reference)	
<25	46/23	0.79 (0.38‐1.67)	138/138	1.34 (0.92‐1.95)	
≥25	42/20	0.84 (0.35‐1.98)	158/203	1.57 (1.08‐2.30)*	0.121
*P*_‐trend_ c		0.644		0.021	
Cumulative amount (pack‐years)					
Never	92/58	1.00 (reference)	244/256	1.00 (reference)	
<30	63/32	0.82 (0.41‐1.64)	192/207	1.40 (0.98‐1.99)	
≥30	27/11	0.59 (0.22‐1.60)	101/136	1.60 (1.05‐2.42)*	0.066
*P*_‐trend_ c		0.305		0.028	
Inhaled smoking or not					
Never	92/58	1.00 (reference)	244/256	1.00 (reference)	
Noninhaled	15/12	1.24 (0.47‐3.29)	68/95	1.77 (1.10‐2.52)**	
Inhaled	64/27	0.71 (0.34‐1.50)	211/237	1.41 (0.99‐2.02)	0.120
*P*_‐trend_ c		0.349		0.121	

a Low level refers to AER < 50% and high EBV level refers to AER ≥ 50%.

b Adjusted for age (continuous variable), sex, education level, drinking, tea consumption, salted fish intake, preserved vegetable intake, herbal tea intake, Canton soup intake.

c Linear trends tests were performed by treating ordered categorical variables as continuous variables.

d *P* values for multiplicative interaction analysis.

* *P* < 0.05.

** *P* < 0.01.

We further explored the potential risk factors associated with the clinical stage of NPC at diagnosis. We found only education level and smoking were significantly associated with clinical stage of NPC at diagnosis among all investigated factors in multivariate analysis. Patients with a higher education level was less likely to receive an advanced NPC diagnosis (OR = 0.47, 95% CI: 0.30‐0.72). Compared with never smokers, current smokers were associated with a higher risk of advanced NPC diagnosis (OR = 2.17, 95% CI: 1.47‐3.22) (Table 6). Further subgroup analysis suggested that smoking was associated with higher risk of advanced NPC diagnosis, with dose‐response effects. Compared with never smokers, smokers with younger initial age of smoking, longer smoking history, greater cumulative amounts of smoking (pack‐years), and noninhaled type of smoking had higher risk of being diagnosed with advanced NPC (Table 7).

**Table 6:** Associations of potential factors with clinical stage at diagnosis in 1701 NPC patients

Variable	NPC stage at diagnosis, cases (%)		Crude OR (95% CI)	Adjusted OR (95% CI) a
	I‐II stage	III‐IV stage		
Age				
<45	169 (20.61)	651 (79.39)	1.00 (reference)	1.00 (reference)
≥45	159 (18.05)	722 (81.95)	1.18 (0.93‐1.50)	1.04 (0.80‐1.36)
Sex				
Female	92 (20.22)	363 (79.78)	1.00 (reference)	1.00 (reference)
Male	236 (18.94)	1010 (81.06)	1.08 (0.83‐1.42)	0.87 (0.61‐1.26)
Education				
Primary school or less	59 (14.86)	338 (85.14)	1.00 (reference)	1.00 (reference)
High school	189 (18.19)	850 (81.81)	0.79 (0.57‐1.08)	0.78 (0.56‐1.11)
University or more	77 (29.84)	77 (29.84)	0.41 (0.28‐0.60)***	0.47 (0.30‐0.72)***
*P*_‐trend_^b^			<0.001	<0.001
Smoking status				
Never smoker	173 (22.56)	594 (77.44)	1.00 (reference)	1.00 (reference)
Ex‐smoker	91 (20.54)	352 (79.46)	1.13 (0.85‐1.50)	1.22 (0.84‐1.75)
Current smoker	61 (12.71)	419 (87.29)	2.00 (1.46‐2.75)***	2.17 (1.47‐3.22)***
*P*_‐trend_ b			<0.001	<0.001
Alcohol drinking				
Nondrinker	198 (20.43)	771 (79.51)	1.00 (reference)	1.00 (reference)
≤1 drink per day	76 (18.72)	330 (81.28)	1.12 (0.83‐1.50)	1.08 (0.78‐1.49)
>1 drink per day	50 (16.13)	260 (83.87)	1.34 (0.95‐1.88)	1.12 (0.76‐1.65)
*P*_‐trend_ b			0.091	0.613
Tea consumption				
Less than monthly	101 (16.97)	494 (83.03)	1.00 (reference)	1.00 (reference)
Monthly	63 (22.18)	221 (77.82)	0.71 (0.50‐1.02)	0.74 (0.51‐1.07)
Weekly or more	156 (19.67)	637 (80.33)	0.83 (0.63‐1.10)	0.75 (0.55‐1.03)
*P*_‐trend_ b			0.239	0.056
Salted fish intake				
Less than monthly	273 (20.09)	1086 (79.91)	1.00 (reference)	1.00 (reference)
Monthly	36 (19.67)	147 (80.33)	1.03 (0.70‐1.51)	0.85 (0.56‐1.31)
Weekly or more	19 (12.75)	130 (87.25)	1.72 (1.04‐2.83)*	1.55 (0.91‐2.63)
*P*_‐trend_ b			0.055	0.245
Preserved vegetable intake				
Less than monthly	250 (20.51)	969 (79.49)	1.00 (reference)	1.00 (reference)
Monthly	35 (13.83)	218 (86.17)	1.61 (1.10‐2.36)*	1.48 (0.98‐2.25)
Weekly or more	43 (19.28)	180 (80.72)	1.08 (0.75‐1.55)	0.91 (0.61‐1.34)
*P*_‐trend_ b			0.218	0.943
Herbal tea intake				
Less than monthly	84 (19.35)	350 (80.65)	1.00 (reference)	1.00 (reference)
Monthly	136 (20.33)	533 (79.67)	0.94 (0.69‐1.27)	0.94 (0.68‐1.30)
Weekly or more	105 (17.89)	482 (82.11)	1.10 (0.80‐1.51)	1.04 (0.74‐1.46)
*P*_‐trend_ b			0.501	0.690
Canton soup intake				
Less than monthly	46 (10.25)	193 (80.75)	1.00 (reference)	1.00 (reference)
Monthly	31 (16.15)	161 (83.85)	1.24 (0.75‐2.04)	1.14 (0.67‐1.93)
Weekly or more	246 (19.62)	1008 (80.38)	0.98 (0.69‐1.39)	0.92 (0.63‐1.34)
*P*_‐trend_ b			0.642	0.562
Family history of NPC				
No	275 (19.43)	1140 (80.57)	1.00 (reference)	1.00 (reference)
Yes	53 (18.79)	229 (81.21)	1.04 (0.75‐1.44)	0.98 (0.70‐1.37)
Family history of tumor^c^				
No	179 (17.99)	816 (82.01)	1.00 (reference)	1.00 (reference)
Yes	149 (21.23)	553 (78.77)	0.81 (0.64‐1.04)	——

a Adjusted for age (<45, ≥45), education level, smoking status, drinking, tea consumption, salted fish intake, preserved vegetable intake, herbal tea intake, Canton soup intake, family history of NPC.

b Linear trends tests were performed by treating ordered categorical variables as continuous variables.

c Family history of tumor was not included in the multivariate analysis.

* *P* < 0.05.

*** *P* < 0.001.

**Table 7:** Subgroup analysis of smoking with NPC stage at diagnosis

Variable	Current smoker				Ex‐smoker			
	I‐II stage	III‐IV stage	OR (95% CI)^a^	*P*‐value^a^	I‐II stage	III‐IV stage	OR (95% CI)^a^	*P*‐value^a^
Initial age of smoking (years)								
Never	173 (22.56)	594 (77.44)	1.00 (reference)	/	173 (22.56)	594 (77.44)	1.00 (reference)	/
≥20	33 (14.73)	191 (85.27)	2.00 (1.24‐3.23)	0.005	53 (23.14)	176 (76.86)	1.00 (0.66‐1.53)	0.986
<20	28 (10.94)	228 (89.06)	2.67 (1.60‐4.44)	<0.001	38 (17.76)	176 (82.24)	1.45 (0.91‐2.33)	0.120
*P_‐_*_trend_ b				<0.001				0.146
Smoking duration (years)								
Never	173 (22.56)	594 (77.44)	1.00 (reference)	/	173 (22.56)	594 (77.44)	1.00 (reference)	/
<25	27 (13.57)	172 (86.43)	2.23 (1.33‐3.74)	0.002	53 (25.00)	159 (75.00)	0.98 (0.64‐1.50)	0.930
≥25	31 (11.48)	239 (88.52)	2.53 (1.52‐4.20)	<0.001	36 (16.22)	186 (83.78)	1.63 (0.99‐2.66)	0.053
*P_‐_*_trend_ b				<0.001				0.082
Cumulative amount (pack‐years)								
Never	173 (22.56)	594 (77.44)	1.00 (reference)	/	173 (22.56)	594 (77.44)	1.00 (reference)	/
<30	37 (12.71)	254 (87.29)	2.37 (1.49‐3.75)	<0.001	68 (23.53)	221 (76.47)	1.07 (0.72‐1.58)	0.744
≥30	22 (12.22)	158 (87.78)	2.38 (1.34‐4.23)	0.003	21 (14.58)	123 (85.42)	1.82 (1.01‐3.28)	0.047
*P_‐_*_trend_ b				<0.001				0.082
Inhaled smoking or not								
Never	173 (22.56)	594 (77.44)	1.00 (reference)	/	173 (22.56)	594 (77.44)	1.00 (reference)	/
Noninhaled	11 (11.46)	85 (88.54)	2.67 (1.32‐5.38)	0.006	23 (17.29)	110 (82.71)	1.51 (0.87‐2.58)	0.140
Inhaled	44 (12.50)	308 (87.50)	2.34 (1.50‐3.66)	<0.001	56 (20.29)	220 (79.71)	1.19 (0.79‐1.83)	0.417
*P_‐_*_trend_ b				<0.001				0.383

a Adjusted for age (<45, ≥45), education level, smoking status, drinking, tea consumption, salted fish intake, preserved vegetable intake, herbal tea intake, Canton soup intake, family history of NPC.

b Linear trends tests were performed by treating ordered categorical variables as continuous variables.

### 3.5 EBV antibodies and other lifestyle factors

Consumption of salted fish and preserved vegetables were found in positive relationships with elevated serum antibody levels of EA‐IgA in univariate analysis, but the associations vanished after adjusting for potential confounding factors. No associations were detected between alcohol drinking, tea consumption, Cantonese slow‐cooked soup, and either of the three EBV antibodies (Table 3).

## 4 DISCUSSION

The role of EBV reactivation in the pathogenesis and progression of NPC has long been established, but the potential risk factors for elevated serological EBV antibodies have not yet been systematically described among NPC patients. In this study, we investigated several potential risk factors for three widely used EBV antibodies namely VCA‐IgA, EA‐IgA, and EBV DNase‐specific neutralizing antibody among NPC patients from South China. We found consumption of Canton‐style herbal tea was consistently in significant association with pretreatment serological VCA‐IgA and EA‐IgA among NPC patients. Weekly consumption of herbal tea could increase the risk of higher pretreatment VCA‐IgA and EA‐IgA, and smoking was identified as a risk factor for EBV DNase‐specific neutralizing antibody among patients with advanced NPC.

Herbal tea is a kind of drink widely consumed in NPC‐endemic areas of Guangdong that contains complex traditional Chinese medicinal herbs.^23^ Though herbal medicine has been linked to NPC for decades, its actual effect on NPC remains indistinct since epidemiological results across different populations are inconsistent. Studies conducted in Philippines^28^, ^29^ have suggested that herbal medicine was significantly associated with increased risk of NPC, and similar associations were also observed in studies carried out in Taiwan^30^ and Guangxi^31^ populations. In contrast, we previously observed a reverse relationship between Canton‐style herbal tea consumption and NPC risk in studies conducted in South China,^21^, ^23^ while Yu et al^32^, ^33^, ^34^ reported a lack of independent association between herbal medicine use and risk of NPC in Guangzhou population. The possible explanation for this discrepancy may be that herbal medicine is too broad a concept that the involved herbal ingredients could be of great interstudy or introstudy heterogeneity. It is unlikely that all herbal medicines increase risk of NPC. Further, Canton‐style herbal tea investigated in our studies contains limited herbal ingredients and cannot be exactly equivalent to traditional herbal medicine. Whatever, more detailed researches are required to elucidate the actual role of herbal medicine in the pathogenesis of NPC.

In addition to directly study the association of herbal medicine with NPC, another alternative attempt is to explore whether herbal medicine is associated with EBV reactivation in the development of NPC. Several in vitro studies have been conducted to explore the potential function of herbal medicine on EBV reactivation, although it has not yet been clearly elucidated. Early in 1980s, Zeng et al^35^, ^36^ have identified extracts from Chinese medicinal herbs as inducers of EA‐IgA expression in Raji cells and similar results were obtained in nasopharyngeal cells by Furukawa et al^37^ Phorbol esters (TPA), produced by herbal plants of Euphorbiaceae growing specially in South China that are usually used as traditional Chinese herbal medicine, has been reported to induce EBV reactivation via NF‐kB and AP‐1 as regulated by PKC and MAPK.^38^ In addition, experiment evidence has suggested that the EBV transformation of normal human epithelial cells depends on the presence of TPA.^39^ These findings indicate that the interaction between herbal medicine and EBV is biologically plausible and herbal medicine might involve in the pathogenesis of NPC either though reactivating EBV or through a promotional effect on cells transformed by EBV. However, we have observed a positive association between monthly consumption of Canton‐style herbal tea and oral EBV DNA loads in healthy population.^25^ In this study, we further validated the association by identifying the positive relationships between consumption of Canton‐style herbal tea and serum VCA‐IgA and EA‐IgA antibodies in NPC patients, especially in older patients, females, those less educated and those with a family history of tumors. Our results add to the evidence for the hypothesis that herbal tea may increase the risk of NPC by promoting the reactivation of EBV. However, conclusions need to be taken with caution given the lack of similar associations between herbal tea and serological antibodies of VCA‐IgA, EBNA1‐IgA, and Zta‐IgA in healthy population in our previous studies.^21^, ^22^ Our results are somewhat consistent with an early study conducted by Hildesheim et al^28^ in the Filipino population, wherein herbal medicine use was found to be correlated with elevated anti‐EBV antibody titers among NPC cases but not among control subjects. Of particular note is that they further observed an apparent interaction between herbal medicine use and anti‐EBV antibodies to NPC risk. These findings led them to claim that herbal medicine interacted with EBV in the development of NPC rather through a promotional effect on EBV‐transformed cells than through inducing EBV reactivation. However, they declared at the same time that their results should be interpreted with special caution due to the small sample size (104 cases and 205 controls). Given that the mechanism underlying the interaction between herbal medicine and EBV in the pathogenesis of NPC remains largely unknown, more in‐depth analysis based on larger epidemiological studies and more detailed molecular cytology studies are needed to elucidate this.

Though the association between cigarette smoking and NPC has been well established by large epidemiological studies in different regions,^15^, ^24^, ^29^, ^34^, ^40^, ^41^, ^42^, ^43^, ^44^, ^45^, ^46^ the underlying mechanisms remain to be further elucidated. Our previous in vitro experiment found cigarette smoke extract could promote EBV lytic gene expression in B cells.^21^ Further, our previous large‐scale multicenter epidemiological study observed positive relationships between smoking and serum seropositivity for VCA‐IgA,^21^ EBNA1‐IgA and Zta‐IgA,^22^ as well as oral EBV DNA loads^25^ among healthy individuals, especially those in areas with high NPC incidence. These results suggest that smoking may increase the potential NPC risk by triggering the EBV reactivation. In this study for NPC patients, we further validated this perspective by identifying a dose‐response positive relationship between smoking and EBV DNase‐specific neutralizing antibody among advanced NPC patients. Additionally, we observed a strong dose‐response relationship between smoking and advanced NPC diagnosis, which was consistent with a previous study.^47^ EBV antibodies have been widely reported to be strongly related to NPC stage,^48^ as observed in this study. Therefore, the triangle relation among cigarette smoking, EBV antibodies, and NPC stage seemed particularly interesting and complicated to interpret. Smoking might raise the risk of advanced NPC though promoting the reactivation of EBV or smoking directly induced advanced NPC and thus resulting in a higher level of EBV reactivation. In other words, the exact location of smoking and EBV in the pathogenic chain of NPC remains unclear and is worthy of further in‐depth exploration in future studies. Besides, another interesting finding in our analysis was that noninhalation of smoke was found to be a stronger risk factor for both higher EBV DNase‐specific neutralizing antibody and advanced NPC diagnosis than inhaled smoking. We suppose that smoking without inhaling deeply allows more smoke to linger in the oropharynx and nasopharynx and may thus increase the risk for NPC incidence. However, this superficial finding requires further in‐depth verification in future studies.

This study is one of the few epidemiological studies that have described in detail the associations of environmental risk factors with serological EBV antibodies among NPC patients. We identified Canton‐style herbal tea and smoking as an independent risk factor for higher serological EBV antibody levels, which is not only crucial for understanding the mechanisms of EBV reactivation in NPC but is also of significance for primary prevention of NPC due to EBV reactivation in South China. This study was conducted on the basis of a well‐designed program with strict quality control for data collection and laboratory testing to ensure the precision of the results. It must be admitted that this study did have some limitations. We did not collect more detailed information on the herbal tea consumption, such as types of herbal tea consumed, the initiation age and duration years of herbal tea intakes, which limited us to further explore the dose‐relationship more precisely in subgroup analysis. Besides, since EBV markers evaluated in this study exhibit lower positive rates in healthy population, we did not include EBV antibodies data of healthy individuals in this study, which limited us to carry out a case‐control study to further evaluate the interaction between environmental factors and these EBV antibodies for NPC risk. Finally, further prospective studies are needed to confirm the causal relationship among these environmental factors such as herbal tea intake and smoking, elevated EBV antibodies and NPC development.

In summary, this study identified the risk effect of herbal tea consumption in addition to smoking on EBV reactivation among NPC patients, providing new insight into the pathogenesis mechanisms of NPC from the perspective of EBV reactivation. We consider smoking cessation, reducing or avoiding herbal tea consumption, which is not that hard to achieve, may have the potential to serve as new approaches to decrease EBV reactivation in high‐risk populations, which may be of potential public health significance for the primary prevention of NPC.

## CONFLICT OF INTEREST

The authors declare that the research was conducted in the absence of any commercial or financial relationships that could be construed as a potential conflict of interest.

## DATA AVAILABILITY STATEMENT

The data that support the findings of this study are available from the corresponding author upon reasonable request.

## References

[1] Epstein MA , Achong BG , Barr YM . Virus particles in cultured lymphoblasts from Burkitt's lymphoma. Lancet. 1964;1(7335):702‐703. 14107961 10.1016/s0140-6736(64)91524-7

[2] Plummer M , de Martel C , Vignat J , Ferlay J , Bray F , Franceschi S . Global burden of cancers attributable to infections in 2012: a synthetic analysis. Lancet Glob Health. 2016;4(9):e609‐e616. 27470177 10.1016/S2214-109X(16)30143-7

[3] Babcock GJ , Decker LL , Volk M , Thorley‐Lawson DA . EBV persistence in memory B cells in vivo. Immunity. 1998;9(3):395‐404. 9768759 10.1016/s1074-7613(00)80622-6

[4] Li H , Liu S , Hu J , et al. Epstein‐Barr virus lytic reactivation regulation and its pathogenic role in carcinogenesis. Int J Biol Sci. 2016;12(11):1309‐1318. 27877083 10.7150/ijbs.16564 PMC5118777

[5] Tang L‐L , Chen W‐Q , Xue W‐Q , et al. Global trends in incidence and mortality of nasopharyngeal carcinoma. Cancer Lett. 2016;374(1):22‐30. 26828135 10.1016/j.canlet.2016.01.040

[6] Old LJ , Boyse EA , Oettgen HF , et al. Precipitating antibody in human serum to an antigen present in cultured Burkitt's lymphoma cells. Proc Natl Acad Sci USA. 1966;56(6):1699‐1704. 16591407 10.1073/pnas.56.6.1699 PMC220158

[7] Chua M , Wee J , Hui EP , Chan A . Nasopharyngeal carcinoma. Lancet. 2016;387(10022):1012‐1024. 26321262 10.1016/S0140-6736(15)00055-0

[8] Chien Y‐C , Chen J‐Y , Liu M‐Y , et al. Serologic markers of Epstein‐Barr virus infection and nasopharyngeal carcinoma in Taiwanese men. N Engl J Med. 2001;345(26):1877‐1882. 11756578 10.1056/NEJMoa011610

[9] Cao S‐M , Liu Z , Jia W‐H , et al. Fluctuations of epstein‐barr virus serological antibodies and risk for nasopharyngeal carcinoma: a prospective screening study with a 20‐year follow‐up. PLoS ONE. 2011;6(4):e19100. 21544243 10.1371/journal.pone.0019100 PMC3081347

[10] Zeng Y , Zhong JM , Li LY , et al. Follow‐up studies on Epstein‐Barr virus IgA/VCA antibody‐positive persons in Zangwu County, China. Intervirology. 1983;20(4):190‐194. 6317603 10.1159/000149391

[11] Zeng Y , Zhang LG , Wu YC , et al. Prospective studies on nasopharyngeal carcinoma in Epstein‐Barr virus IgA/VCA antibody‐positive persons in Wuzhou City, China. Int J Cancer. 1985;36(5):545‐547. 4055129 10.1002/ijc.2910360505

[12] Zeng Y , Jan MG , Zhang Q , et al. Serological mass survey for early detection of nasopharyngeal carcinoma in Wuzhou City, China. Int J Cancer. 1982;29(2):139‐141. 6895884 10.1002/ijc.2910290204

[13] Deng H , Zeng Y , Lei Y , et al. Serological survey of nasopharyngeal carcinoma in 21 cities of south China. Chin Med J (Engl). 1995;108(4):300‐303. 7789220

[14] Liu Y , Huang Q , Liu W , et al. Establishment of VCA and EBNA1 IgA‐based combination by enzyme‐linked immunosorbent assay as preferred screening method for nasopharyngeal carcinoma: a two‐stage design with a preliminary performance study and a mass screening in southern China. Int J Cancer. 2012;131(2):406‐416. 21866545 10.1002/ijc.26380

[15] Hu T , Lin C‐Y , Xie S‐H , et al. Smoking can increase nasopharyngeal carcinoma risk by repeatedly reactivating Epstein‐Barr Virus: an analysis of a prospective study in southern China. Cancer Med. 2019. 10.1002/cam4.2083 PMC6536979 30843658

[16] Ji M‐F , Yu Y‐L , Cheng W‐M , et al. Detection of stage I nasopharyngeal carcinoma by serologic screening and clinical examination. Chin J Cancer. 2011;30(2):120‐123. 21272444 10.5732/cjc.010.10595 PMC4013341

[17] Xu J , Wan X‐B , Huang X‐F , et al. Serologic antienzyme rate of Epstein‐Barr virus DNase‐specific neutralizing antibody segregates TNM classification in nasopharyngeal carcinoma. J Clin Oncol. 2010;28(35):5202‐5209. 21060035 10.1200/JCO.2009.25.6552

[18] De‐Vathaire F , Sancho‐Garner H , De‐Thé H , et al. Prognostic value of EBV markers in the clinical management of nasopharyngeal carcinoma (NPC): a multicenter follow‐up study. Int J Cancer. 1988;42(2):176‐181. 2841245 10.1002/ijc.2910420206

[19] Liu MT , Yeh CY . Prognostic value of anti‐Epstein‐Barr virus antibodies in nasopharyngeal carcinoma (NPC). Radiat Med. 1998;16(2):113‐117. 9650898

[20] Zhao F‐P , Liu X , Zhong Z‐M , et al. Positivity of both plasma Epstein‐Barr virus DNA and serum Epstein‐Barr virus capsid specific immunoglobulin A is a better prognostic biomarker for nasopharyngeal carcinoma. BBA Clin. 2014;2:88‐93. 26673151 10.1016/j.bbacli.2014.10.003 PMC4655226

[21] Xu F‐H , Xiong D , Xu Y‐F , et al. An epidemiological and molecular study of the relationship between smoking, risk of nasopharyngeal carcinoma, and Epstein‐Barr virus activation. J Natl Cancer Inst. 2012;104(18):1396‐1410. 22972969 10.1093/jnci/djs320

[22] He Y‐Q , Xue W‐Q , Xu F‐H , et al. The relationship between environmental factors and the profile of Epstein‐Barr virus antibodies in the lytic and latent infection periods in healthy populations from endemic and non‐endemic nasopharyngeal carcinoma areas in China. EBioMedicine. 2018;30:184‐191. 29606628 10.1016/j.ebiom.2018.02.019 PMC5952216

[23] Jia W‐H , Luo X‐Y , Feng B‐J , et al. Traditional Cantonese diet and nasopharyngeal carcinoma risk: a large‐scale case‐control study in Guangdong, China. BMC Cancer. 2010;10:446. 20727127 10.1186/1471-2407-10-446 PMC2931495

[24] Xue WQ , Qin HD , Ruan HL , Shugart YY , Jia WH . Quantitative association of tobacco smoking with the risk of nasopharyngeal carcinoma: a comprehensive meta‐analysis of studies conducted between 1979 and 2011. Am J Epidemiol. 2013;178(3):325‐338. 23785114 10.1093/aje/kws479 PMC3727336

[25] He Y‐Q , Liao X‐Y , Xue W‐Q , et al. Association between environmental factors and oral Epstein‐Barr virus DNA loads: a multicenter cross‐sectional study in China. J Infect Dis. 2019;219(3):400‐409. 30307559 10.1093/infdis/jiy542 PMC6941616

[26] Tang JW , Rohwäder E , Chu I , et al. Evaluation of Epstein‐Barr virus antigen‐based immunoassays for serological diagnosis of nasopharyngeal carcinoma. J Clin Virol. 2007;40(4):284‐288. 17977062 10.1016/j.jcv.2007.09.006

[27] Xue WQ , He YQ , Liao XY , et al. Decreased oral Epstein‐Barr virus DNA loads in patients with nasopharyngeal carcinoma in Southern China: a case‐control and a family‐based study. Cancer Med. 2018;7:3453‐3464. 10.1002/cam4.1597 PMC6051183 29905022

[28] Hildesheim A , West S , DeVeyra E , et al. Herbal medicine use, Epstein‐Barr virus, and risk of nasopharyngeal carcinoma. Cancer Res. 1992;52(11):3048‐3051. 1317256

[29] West S , Hildesheim A , Dosemeci M . Non‐viral risk factors for nasopharyngeal carcinoma in the Philippines: results from a case‐control study. Int J Cancer. 1993;55(5):722‐727. 7503957 10.1002/ijc.2910550504

[30] Lin TM , Chen KP , Lin CC , et al. Retrospective study on nasopharyngeal carcinoma. J Natl Cancer Inst. 1973;51(5):1403‐1408. 4762926 10.1093/jnci/51.5.1403

[31] Zheng YM , Tuppin P , Hubert A , et al. Environmental and dietary risk factors for nasopharyngeal carcinoma: a case‐control study in Zangwu County, Guangxi, China. Br J Cancer. 1994;69(3):508‐514. 8123482 10.1038/bjc.1994.92 PMC1968852

[32] Yu MC , Huang TB , Henderson BE . Diet and nasopharyngeal carcinoma: a case‐control study in Guangzhou, China. Int J Cancer. 1989;43(6):1077‐1082. 2732001 10.1002/ijc.2910430621

[33] Yu MC , Ho JH , Lai SH , Henderson BE . Cantonese‐style salted fish as a cause of nasopharyngeal carcinoma: report of a case‐control study in Hong Kong. Cancer Res. 1986;46(2):956‐961. 3940655

[34] Yu MC , Garabrant DH , Huang TB , Henderson BE . Occupational and other non‐dietary risk factors for nasopharyngeal carcinoma in Guangzhou, China. Int J Cancer. 1990;45(6):1033‐1039. 2351484 10.1002/ijc.2910450609

[35] Zeng Y , Zhong JM , Mo YK , Miao XC . Epstein‐Barr virus early antigen induction in Raji cells by Chinese medicinal herbs. Intervirology. 1983;19(4):201‐204. 6305871 10.1159/000149361

[36] Zeng Y , Zhong JM , Ye SQ , et al. Screening of Epstein‐Barr virus early antigen expression inducers from Chinese medicinal herbs and plants. Biomed Environ Sci. 1994;7(1):50‐55. 8024719

[37] Furukawa M , Komori T , Ishiguro H , Umeda R . Epstein‐Barr virus early antigen induction in nasopharyngeal hybrid cells by Chinese medicinal herbs. Auris Nasus Larynx. 1986;13(2):101‐105. 3028348 10.1016/s0385-8146(86)80005-0

[38] Gao X , Ikuta K , Tajima M , Sairenji T . 12‐O‐tetradecanoylphorbol‐13‐acetate induces Epstein‐Barr virus reactivation via NF‐kappaB and AP‐1 as regulated by protein kinase C and mitogen‐activated protein kinase. Virology. 2001;286(1):91‐99. 11448162 10.1006/viro.2001.0965

[39] Tomei LD , Noyes I , Blocker D , Holliday J , Glaser R . Phorbol ester and Epstein‐Barr virus dependent transformation of normal primary human skin epithelial cells. Nature. 1987;329(6134):73‐75. 2442617 10.1038/329073a0

[40] Hsu W‐L , Chen J‐Y , Chien Y‐C , et al. Independent effect of EBV and cigarette smoking on nasopharyngeal carcinoma: a 20‐year follow‐up study on 9,622 males without family history in Taiwan. Cancer Epidemiol Biomarkers Prev. 2009;18(4):1218‐1226. 19336547 10.1158/1055-9965.EPI-08-1175

[41] Nam J‐M , McLaughlin JK , Blot WJ . Cigarette smoking, alcohol, and nasopharyngeal carcinoma: a case‐control study among U.S. whites. J Natl Cancer Inst. 1992;84(8):619‐622. 1556772 10.1093/jnci/84.8.619

[42] Chow WH , McLaughlin JK , Hrubec Z , Nam JM , Blot WJ . Tobacco use and nasopharyngeal carcinoma in a cohort of US veterans. Int J Cancer. 1993;55(4):538‐540. 8406978 10.1002/ijc.2910550403

[43] Vaughan TL , Shapiro JA , Burt RD , et al. Nasopharyngeal cancer in a low‐risk population: defining risk factors by histological type. Cancer Epidemiol Biomarkers Prev. 1996;5(8):587‐593. 8824359

[44] Yuan JM , Wang XL , Xiang YB , Gao YT , Ross RK , Yu MC . Non‐dietary risk factors for nasopharyngeal carcinoma in Shanghai, China. Int J Cancer. 2000;85(3):364‐369. 10652428

[45] Cheng YJ , Hildesheim A , Hsu MM , et al. Cigarette smoking, alcohol consumption and risk of nasopharyngeal carcinoma in Taiwan. Cancer Causes Control. 1999;10(3):201‐207. 10454065 10.1023/a:1008893109257

[46] Armstrong RW , Imrey PB , Lye MS , Armstrong MJ , Yu MC , Sani S . Nasopharyngeal carcinoma in Malaysian Chinese: occupational exposures to particles, formaldehyde and heat. Int J Epidemiol. 2000;29(6):991‐998. 11101539 10.1093/ije/29.6.991

[47] Ren JT , Li MY , Wang XW , Xue WQ , Ren ZF , Jia WH . Potential factors associated with clinical stage of nasopharyngeal carcinoma at diagnosis: a case‐control study. Chin J Cancer. 2017;36(1):71. 28870229 10.1186/s40880-017-0239-y PMC5584009

[48] Shao J‐Y , Li Y‐H , Gao H‐Y , et al. Comparison of plasma Epstein‐Barr virus (EBV) DNA levels and serum EBV immunoglobulin A/virus capsid antigen antibody titers in patients with nasopharyngeal carcinoma. Cancer‐Am Cancer Soc. 2004;100(6):1162‐1170. 10.1002/cncr.20099 15022282

