# Supplementary material for: Associations between environmental factors and serological Epstein‐Barr virus antibodies in patients with nasopharyngeal carcinoma in South China
Source: Cancer Med. 2019 Jun 26;8(10):4852–66. doi: 10.1002/cam4.2348 (PMC6712476; doi:10.1002/cam4.2348)
Supplement: Supplementary file 1 [file CAM4-8-4852-s001.docx]

**Table S1** **Subgroup analysis of the associations between herbal tea consumption and serological antibodies of VCA-IgA and EA-IgA**

| Subgroups | VCA-IgA | | |  | EA-IgA | | |
| --- | --- | --- | --- | --- | --- | --- | --- |
|  | Low / High^†^ | OR (95%CI) ^‡^ | *P-*value |  | Low / High^†^ | OR (95%CI) ^‡^ | *P-*value |
| **Age, years** |  |  |  |  |  |  |  |
| **<40** |  |  |  |  |  |  |  |
| Less than monthly | 62 / 54 | 1.00 (reference) | / |  | 68 / 48 | 1.00 (reference) | / |
| Monthly | 106 / 104 | 1.15 (0.71-1.85) | 0.565 |  | 129 / 81 | 0.86 (0.53-1.41) | 0.558 |
| Weekly or more | 85 / 77 | 1.09 (0.65-1.82) | 0.748 |  | 95 / 67 | 1.07 (0.63-1.80) | 0.808 |
| *P*_-trend_ ^§^ |  |  | 0.784 |  |  |  | 0.739 |
| **≥40** |  |  |  |  |  |  |  |
| Less than monthly | 156 / 161 | 1.00 (reference) | / |  | 179 / 138 | 1.00 (reference) | / |
| Monthly | 204 / 254 | 1.14 (0.84-1.55) | 0.405 |  | 248 / 209 | 1.07 (0.79-1.45) | 0.666 |
| Weekly or more | 162 / 259 | 1.41 (1.03-1.94) | 0.034 |  | 98 / 223 | 1.38 (1.01-1.89) | 0.046 |
| *P*_-trend_ ^§^ |  |  | 0.031 |  |  |  | 0.037 |
| *P*-interaction ^¶^ |  |  | 0.184 |  |  |  | 0.233 |
| **Sex** |  |  |  |  |  |  |  |
| **Female** |  |  |  |  |  |  |  |
| Less than monthly | 79 / 60 | 1.00 (reference) | / |  | 81 / 58 | 1.00 (reference) | / |
| Monthly | 87 / 106 | 1.93 (1.19-3.13) | 0.007 |  | 110 / 82 | 1.12 (0.69-1.80) | 0.654 |
| Weekly or more | 52 / 66 | 1.87 (1.09-3.23) | 0.024 |  | 60 / 58 | 1.41 (0.82-2.42) | 0.210 |
| *P*_-trend_ ^§^ |  |  | 0.020 |  |  |  | 0.215 |
| **Male** |  |  |  |  |  |  |  |
| Less than monthly | 139 / 155 | 1.00 (reference) | / |  | 166 / 128 | 1.00 (reference) | / |
| Monthly | 223 / 252 | 0.96 (0.71-1.31) | 0.802 |  | 267 / 208 | 0.98 (0.72-1.34) | 0.898 |
| Weekly or more | 195 / 270 | 1.17 (0.85-1.60) | 0.329 |  | 233 / 232 | 1.28 (0.94-1.76) | 0.117 |
| *P*_-trend_ ^§^ |  |  | 0.255 |  |  |  | 0.075 |
| *P*-interaction ^¶^ |  |  | 0.313 |  |  |  | 0.995 |
| **Education level** |  |  |  |  |  |  |  |
| **High school or less** |  |  |  |  |  |  |  |
| Less than monthly | 179 / 182 | 1.00 (reference) | / |  | 202 / 159 | 1.00 (reference) | / |
| Monthly | 255 / 303 | 1.17 (0.88-1.55) | 0.279 |  | 319 / 238 | 0.94 (0.71-1.25) | 0.689 |
| Weekly or more | 200 / 300 | 1.44 (1.07-1.93) | 0.015 |  | 241 / 259 | 1.38 (1.03-1.85) | 0.032 |
| *P*_-trend_ ^§^ |  |  | 0.014 |  |  |  | 0.017 |
| **University or more** |  |  |  |  |  |  |  |
| Less than monthly | 38 / 32 | 1.00 (reference) | / |  | 43 / 27 | 1.00 (reference) | / |
| Monthly | 54 / 53 | 1.29 (0.58-2.03) | 0.461 |  | 56 / 51 | 1.71 (0.86-2.42) | 0.129 |
| Weekly or more | 46 / 35 | 0.86 (0.44,1.67) | 0.787 |  | 51 / 30 | 0.97 (0.47-2.00) | 0.933 |
| *P*_-trend_ ^§^ |  |  | 0.774 |  |  |  | 0.903 |
| *P*-interaction ^¶^ |  |  | 0.160 |  |  |  | 0.212 |
| **Clinical stage** |  |  |  |  |  |  |  |
| **I~II** |  |  |  |  |  |  |  |
| Less than monthly | 60 / 23 | 1.00 (reference) | / |  | 62 / 21 | 1.00 (reference) | / |
| Monthly | 80 / 56 | 1.79 (0.93-3.43) | 0.081 |  | 94 / 42 | 1.27 (0.64-2.51) | 0.494 |
| Weekly or more | 64 / 41 | 1.56 (0.77-3.15) | 0.213 |  | 72 / 33 | 1.43 (0.69-2.97) | 0.331 |
| *P*_-trend_ ^§^ |  |  | 0.285 |  |  |  | 0.341 |
| **III~IV** |  |  |  |  |  |  |  |
| Less than monthly | 158 / 192 | 1.00 (reference) | / |  | 185 / 165 | 1.00 (reference) | / |
| Monthly | 230 / 302 | 1.10 (0.83-1.45) | 0.529 |  | 283 / 248 | 1.00 (0.75-1.32) | 0.983 |
| Weekly or more | 183 / 295 | 1.28 (0.95-1.72) | 0.100 |  | 221 / 257 | 1.28 (0.95-1.71) | 0.100 |
| *P*_-trend_ ^§^ |  |  | 0.093 |  |  |  | 0.077 |
| *P*-interaction ^¶^ |  |  | 0.436 |  |  |  | 0.624 |
| **Family history of NPC** |  |  |  |  |  |  |  |
| **No** |  |  |  |  |  |  |  |
| Less than monthly | 179 / 182 | 1.00 (reference) | / |  | 208 / 153 | 1.00 (reference) | / |
| Monthly | 261 / 292 | 1.07 (0.80-1.42) | 0.662 |  | 314 / 238 | 1.02 (0.76-1.36) | 0.900 |
| Weekly or more | 211 / 274 | 1.17 (0.87-1.58) | 0.299 |  | 250 / 234 | 1.24 (0.92-1.67) | 0.161 |
| *P*_-trend_ ^§^ |  |  | 0.290 |  |  |  | 0.134 |
| **Yes** |  |  |  |  |  |  |  |
| Less than monthly | 39 / 32 | 1.00 (reference) | / |  | 39 / 32 | 1.00 (reference) | / |
| Monthly | 48 / 65 | 1.70 (0.91-3.19) | 0.097 |  | 63 / 50 | 0.98 (0.52-1.85) | 0.958 |
| Weekly or more | 36 / 61 | 2.21 (1.14-4.28) | 0.019 |  | 43 / 55 | 1.65 (0.85-3.19) | 0.139 |
| *P*_-trend_ ^§^ |  |  | 0.021 |  |  |  | 0.117 |
| *P*-interaction ^¶^ |  |  | 0.145 |  |  |  | 0.495 |
| **Family history of tumor** |  |  |  |  |  |  |  |
| **No** |  |  |  |  |  |  |  |
| Less than monthly | 121 / 137 | 1.00 (reference) | / |  | 141 / 117 | 1.00 (reference) | / |
| Monthly | 182 / 211 | 0.99 (0.71-1.39) | 0.972 |  | 216 / 176 | 0.98 (0.70-1.38) | 0.919 |
| Weekly or more | 141 / 192 | 1.11 (0.77-1.59) | 0.573 |  | 170 / 163 | 1.14 (0.79-1.63) | 0.483 |
| *P*_-trend_ ^§^ |  |  | 0.545 |  |  |  | 0.444 |
| **Yes** |  |  |  |  |  |  |  |
| Less than monthly | 97 / 77 | 1.00 (reference) | / |  | 106 / 68 | 1.00 (reference) | / |
| Monthly | 127 / 146 | 1.51 (1.00-2.27) | 0.048 |  | 161 / 112 | 1.10 (0.73-1.66) | 0.642 |
| Weekly or more | 106 / 143 | 1.90 (1.25-2.89) | 0.003 |  | 123 / 126 | 1.68 (1.11-2.56) | 0.015 |
| *P*_-trend_ ^§^ |  |  | 0.003 |  |  |  | 0.010 |
| *P*-interaction ^¶^ |  |  | 0.100 |  |  |  | 0.095 |

Abbreviations: VCA-IgA, immunoglobulin A antibodies against viral capsid antigen; EA-IgA, immunoglobulin A antibodies against early antigen.

^†^ For VCA-IgA titers, a low level refers to titers <1:320 and a high EBV level refers to ≥1:320; for EA-IgA titers, a low level refers to titers <1:40 and a high EBV level refers to ≥1:40.

^‡^ Adjusted for age (continuous variable), sex, education level, clinical stage, smoking status, drinking, tea consumption, salted fish intake, preserved vegetable intake, Canton soup intake.

^§^ Linear trends tests were performed by treating ordered categorical variables as continuous variables**.**

^¶^ *P* value from multiplicative model.
